# Mutation Status and Immunohistochemical Correlation of *KRAS, NRAS*, and *BRAF* in 260 Chinese Colorectal and Gastric Cancers

**DOI:** 10.3389/fonc.2018.00487

**Published:** 2018-10-26

**Authors:** Qiwei Yang, Sibo Huo, Yujie Sui, Zhenwu Du, Haiyue Zhao, Yu Liu, Wei Li, Xin Wan, Tongjun Liu, Guizhen Zhang

**Affiliations:** ^1^Medical Research Center, The Second Hospital of Jilin University, Changchun, China; ^2^Department of General Surgery, The Second Hospital of Jilin University, Changchun, China; ^3^Department of Orthopedics, The Second Hospital of Jilin University, Changchun, China; ^4^Center of Reproductive Medicine and Center of Prenatal Diagnosis, The First Hospital of Jilin University, Changchun, China

**Keywords:** colorectal cancer, gastric cancer, *KRAS* mutation, *NRAS* mutation, *BRAF* mutation, immunohistochemistry

## Abstract

*KRAS, NRAS* and *BRAF* are kinases involved in the RAS-RAF-MAPK signaling pathway and also potential tumor-driven genes. Patients with *KRAS*/*NRAS*/*BRAF* mutations are resistant to anti-*EGFR* monoclonal antibody therapy. The main purpose of this study is to investigate the mutation status and distribution of *KRAS*/*NRAS*/*BRAF* in Chinese colorectal and gastric cancers, and to explore the histopathological changes and related immunohistochemical marker changes caused by these mutations. The mutation status of *KRAS* (exons 2, codon 12/13), *NRAS* (exons 2/3/4, codon 12/13/59/61/117/146) and *BRAF* (exons 15, codon 600) were detected by amplification refractory mutation system polymerase chain reaction (ARMS-PCR) in 86 colon cancer, 140 rectal cancer and 34 gastric cancer tissues. Then, the frequencies and distribution of *KRAS*/*NRAS*/*BRAF* mutations were described in detail. Furthermore, the relationship between *KRAS*/*NRAS*/*BRAF* mutations and the features of histopathological and related immunohistochemical markers were analyzed. The results showed that *KRAS*/*NRAS*/*BRAF* mutation rates in colon cancer were 44.2, 1.2, and 3.5%; in rectal cancer were 37.1, 4.3, and 0.7%; in gastric cancer were none, none and 2.9%. The mutation rate of *KRAS* in female (48.8%) is significantly higher than that of male (27.8%), and the mutation rate increased with the higher degree of differentiation. Additionally, the mutation rate of *BRAF* detected by ARMS-PCR (1.77%) was significantly lower than that by immunohistochemistry (4.11%). It also showed that the *KRAS*/*NRAS*/*BRAF* mutation status had a certain relationship with the expression of some immunohistochemical markers. This study provides more data support for clinical research on *KRAS*/*NRAS*/*BRAF* mutation in CRCs or gastric cancers.

## Introduction

Colorectal cancer (CRC) and gastric cancer (GC) are common gastrointestinal cancers. The latest epidemiological data shows that the incidence of CRC ranks 4th in malignant tumors, and the mortality rate ranks 2nd; the incidence and mortality of GC both ranks the 16th in malignant tumors (1). Symptoms of CRC and GC are occult, most patients are diagnosed until advanced stages. According to statistics from the National Cancer Institute (https://seer.cancer.gov/statfacts/), the 5-year survival rate is 64.5% for CRC and 31.0% for GC under current treatment conditions (2). In recent years, the advent of anti-epidermal growth factor receptor (*EGFR*) monoclonal antibodies (MoAbs), such as cetuximab and panitumumab, have contributed to improving the 5-year survival of CRC patients. The benefits of individual genetic profiling for the selection of therapy have been proven in clinical use. However, the incidence and mortality of CRC and GC remain high.

The main function of anti-*EGFR* MoAbs is to compete with endogenous ligands for binding to *EGFR*, thereby blocking downstream RAS and MAPK signaling pathways, inhibiting proliferation of cancer cells, and prolonging the survival of patients with advanced cancer (3). *KRAS, NRAS* and *BRAF* are kinases on the RAS-RAF-MAPK signaling pathway. If the RAS and RAF genes are mutated, the mutated protein will not be regulated by the upstream *EGFR* signal and remain in the activated state, continuing to activate the downstream MAPK pathway, leading to cell uncontrolled proliferation and canceration (4). What's worse, mutations in the RAS and RAF genes are independent of each other, and mutations in either of them will lead to activation of the RAS-RAF-MAPK signaling pathway. Meanwhile, *KRAS, NRAS* and *BRAF* are potential tumor-driven genes themselves (5). Therefore, only patients with wild-type *KRAS, NRAS*, and *BRAF* genes can benefit from anti-*EGFR* targeted therapy (6–8), while patients with *KRAS, NRAS*, and *BRAF* mutations are resistant to anti-*EGFR* MoAbs therapy (9). Detection of *KRAS, NRAS*, and *BRAF* gene mutation status in CRC tissue is a direct and effective method for screening patients for using anti-*EGFR* targeted drugs (10). The 2017 edition of National Comprehensive Cancer Network (NCCN) recommends that *KRAS, NRAS*, and *BRAF* gene mutations should be identified in primary or metastatic tumors of patients with metastatic colorectal cancer, as a basis for predicting whether or not the patient should be treated with anti-*EGFR* MoAbs (11). Therefore, the detection of multiple genes such as *KRAS, NRAS*, and *BRAF* can accurately predict the efficacy of anti-*EGFR* MoAbs, thereby realizing individualized targeted therapy.

98.5% of the *KRAS* mutation occurs in codons 12 or 13 of exon 2. The common mutation site of *NRAS* gene is located in exons 2, 3, and 4 (6). About 81.9% *BRAF* mutations are located at codon 600 with a conversion of valine to glutamic acid (V600E) (4). Several studies indicated that different mutation types of *KRAS, NRAS*, and *BRAF* gene in colorectal cancer tissues have different biological characteristics and lead to different biological changes, which may have different effects on patients. For example, a growing number of studies have shown that patients with a mutation in codon 13 of the *KRAS* gene may have a poorer prognosis but may significantly benefit from an anti-*EGFR* targeted therapy (12). However, some other studies have denied this conclusion. Apparently, the effects of different mutations on the biological properties of tumors and the real mechanisms that lead to different outcomes need to be further elucidated. Most of the previous studies focused on the frequencies and prognostic values of *KRAS, NRAS*, and *BRAF* mutations, however, there is still a lack of understanding of the histopathological changes and other related protein expressions changes caused by these mutations. At the same time, the *KRAS, NRAS*, and *BRAF* gene mutation status and the related histopathological changes in GC tissue is still rarely reported.

In the present study, firstly, we detected the common mutation sites of *KRAS, NRAS*, and *BRAF* gene in CRC and GC tissues of 260 patients by amplification-refractory mutation system polymerase chain reaction (ARMS-PCR). Then, we investigated the frequencies and biological characteristics of *KRAS, NRAS*, and *BRAF* mutations. Subsequently, we analyzed the relationship between *KRAS, NRAS*, and *BRAF* mutations and the changes of histopathological features and related protein expressions. In order to better explain the potential effect of *KRAS, NRAS*, and *BRAF* mutations on the efficacy of anti-*EGFR* MoAbs targeted therapy and the prognosis of CRC and GC patients.

## Materials and methods

### Samples

Two hundred sixty patients (including 86 cases of colon cancer, 140 cases of rectal cancer and 34 cases of gastric cancer) are consecutively collected at the Second Hospital of Jilin University between November 2016 and June 2018. All cases were diagnosed as CRC or GC by two independent pathologists. For each sample, the histopathological sections were stained by hematoxylin-eosin (HE) and immunohistochemistry (IHC) for clinical pathological diagnosis. No patients had accepted preoperative adjuvant treatment. The patients' information is listed in Table 1.

**Table 1:** Clinicopathological characteristics of the patients.

**Factor**	**Colon cancer (*n* = 86), *n* (%)**	**Rectal cancer (*n* = 140), *n* (%)**	**Gastric cancer (*n* = 34), *n* (%)**	**Total (*n* = 260), *n* (%)**
**AGE (YEARS)**'				
Mean ± SD	63.53 ± 11.24	61.53 ± 10.39	64.09 ± 8.74	62.47 ± 10.51
Median	64.5 (range, 29–88)	62 (range, 35–87)	64 (range, 49–83)	63 (range, 29–88)
<60	28 (32.6)	58 (41.4)	10 (29.4)	96 (36.9)
≥60	58 (67.4)	82 (58.6)	24 (70.6)	164 (63.1)
**SEX**				
Male	51 (59.3)	99 (70.7)	26 (76.5)	176 (67.7)
Female	35 (40.7)	41 (29.3)	8 (23.5)	84 (32.3)
**HISTOLOGICAL GRADE**				
I	2 (2.3)	25 (17.9)	2 (5.9)	29 (11.2)
II	42 (48.8)	38 (27.1)	3 (8.8)	83 (31.9)
III	40 (46.5)	77 (55.0)	12 (35.3)	129 (49.6)
IV	2 (2.3)	0 (0)	17 (50.0)	19 (7.3)
**NODAL STATUS**				
Positive	42 (48.8)	77 (55.0)	30 (88.2)	149 (57.3)
Negative	44 (51.2)	63 (45.0)	4 (11.8)	111 (42.7)

### Ethics statement

The Ethics Committee of the Second Hospital of Jilin University has a detailed understanding of and approved all experimental protocols in this study. This study conforms to the provisions of the Declaration of Helsinki (as revised in Fortaleza, Brazil, October 2013). We informed all participants according to the consent for the use of their specimens, and written consents were obtained from each patient. All involved methods were carried out in accordance with relevant guidelines and regulations of the Ethics Committee of the Second Hospital of Jilin University.

### DNA extraction and mutation detection

Genomic DNA was extracted from surgically excised fresh solid tumor tissues. The TIANamp Genomic DNA Kit (Tiangen Biotech, Beijing, China) were used according to the manufacturer's instructions.

For each sample, mutations of *KRAS* exons 2 (codon 12 and 13) were detected by Human *KRAS* Gene Seven Mutation Detection Kit (YZY Medical Science & Technology Co., Ltd., Wuhan, China); mutations of *NRAS* exons 2 (codon 12 and 13), exons 3 (codon 59 and 61), exons 4 (codon 117 and 146) were detected by Human *NRAS* Gene Mutation Detection Kit (YZY Medical Science & Technology Co., Ltd., Wuhan, China); mutations of *BRAF* exons 15 (codon 600) was detected by Human *BRAF* Gene V600E Mutation Detection Kit (YZY Medical Science & Technology Co., Ltd., Wuhan, China). All operations were strictly performed in accordance with the kit manual. Specifically, diluted 30 ng of total DNA sample to 2 μl, then mixed with 0.2 μl polymerase. The mixture was then added to a tube preloaded with a dual fluorescent probe primer. Real-time quantitative polymerase chain reaction was performed by ABI 7500 Fast Dx (Applied Biosystems Co. Ltd., US) as 37°C for 10 min, 95°C for 5 min, then 40 cycles of 95°C for 15 s and 60°C for 60 s.

### IHC staining

All samples were fixed in 4% neutral formaldehyde solution and embedded in paraffin. Tissue block was sliced into 2 μm and dewaxed, hydrated and antigen repaired by PT link (Dako, Agilent Technologies, USA). Specifically, place the slices in the 65°C preheated repair solution, and incubated for 30 min by heating to 90°C, then cooled to 70°C. Subsequently, the slices were washed by PBS. Primary, secondary antibodies and DAB coloring solution were automated incubated by Autostainer Link 48 (Dako, Agilent Technologies, USA). Specifically, incubated with hydrogen peroxide for 10 min, primary antibody for 30 min, and secondary antibody for 20 min in room temperature. Counterstain with hematoxylin, routine dehydration, transparent, and seal.

### Statistical analysis

Statistical analysis was performed using the SPSS version 21 (SPSS Inc., USA). Categorical variables were compared by the Chi-square or Fisher's exact test; quantitative and ordered variables were compared by the Mann-Whitney test. Normally distributed variables were compared by Student's *t*-test. The correspondence relationship between mutation status and immunohistochemical marker characteristics were analyzed using Canonical Correlation Analysis and Multiple Correspondence Analysis. *P* < 0.05 indicate the statistically significantly difference. The Kaplan-Meier (KM) method were used to evaluate the time to diagnosis of survival, recurrence and metastases.

## Results

### Mutation frequencies and distributions

#### General situation

**Figure 1:**
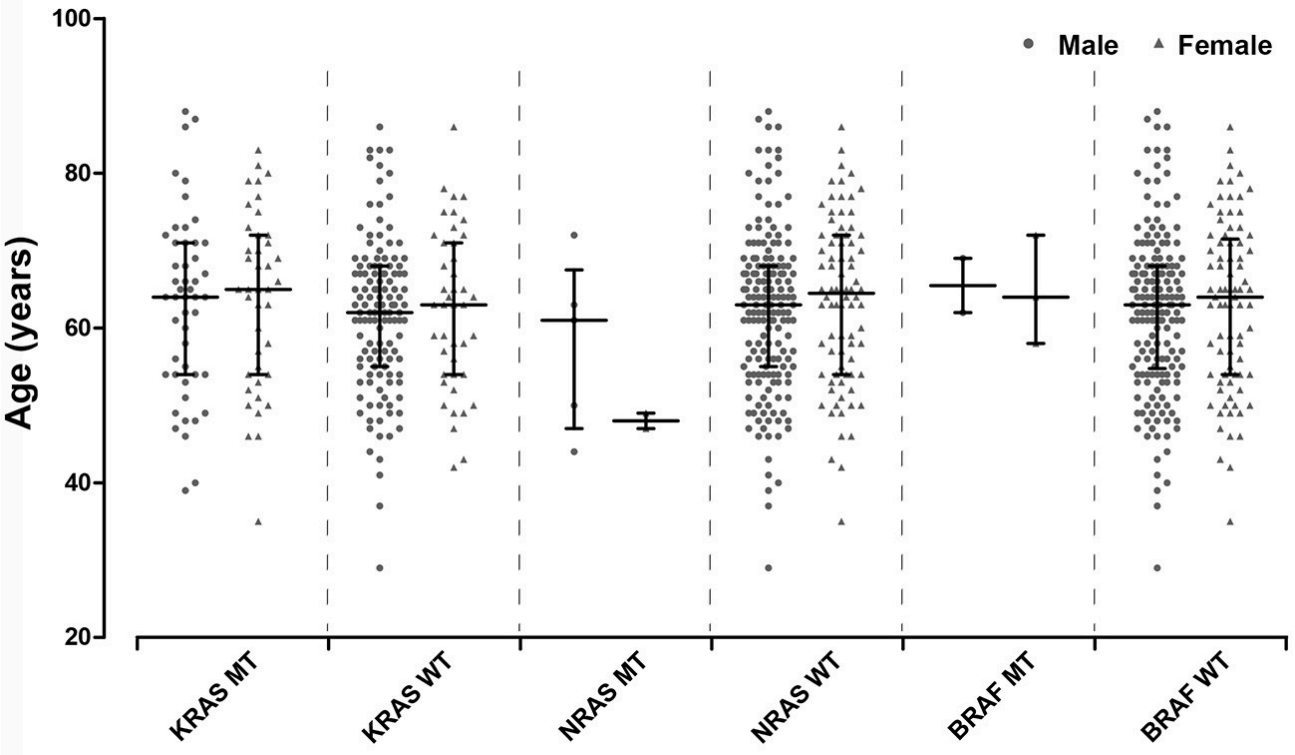
Age distribution of KRAS/NRAS/BRAF mutant type and wild type patients.

The distribution of age between *KRAS*/*NRAS*/*BRAF* mutant type (MT) and wild type (WT) was compared by Student's *t*-test. Additionally, Chi-square test was amplified to analyze the distribution of different age components (divided into two groups by 60 years old) in *KRAS*/*NRAS*/*BRAF* MT and WT. It can be found that *KRAS*/*NRAS*/*BRAF* mutations were not significantly related to patients' age (Figure 1, Table 2). When analyzing the relationship between gender and *KRAS*/*NRAS*/*BRAF* mutations, it can be found that the mutation rate of *KRAS* gene in female (48.8%) is significantly higher than that of male (27.8%) (*p* = 0.001). When analyzing the mutation rates of *KRAS, NRAS*, and *BRAF* in different locations, it can be found that *KRAS* gene mutation rate was significantly different in colon cancers (44.2%), rectal cancers (37.1%) and gastric cancers (0%) (*p* < 0.001). The mutation distribution is shown in Table 2.

**Table 2:** Histopathological characteristics according to KRAS/NRAS/BRAF mutation status.

		**Total Case**	**KRAS (codon 12/13)**			**NRAS (codon 12/13/59/61/117/146)**			**BRAF (codon 600)**		
		**260**	**MT, *n***	**WT, *n***	***p* value**	**MT, *n***	**WT, *n***	***p* value**	**MT, *n***	**WT, *n***	***p*-value**
Age	Mean ± SD		63.46 ± 11.43	61.94 ± 9.98	0.270^†^	55.14 ± 10.29	62.67 ± 10.46	0.062^†^	65.00 ± 5.57	62.42 ± 10.58	0.587^†^
	<60	96	31	65	0.547^‡^	4	92	0.216^‡^	1	95	0.428^‡^
	≥60	164	59	105		3	161		4	160
Sex	Male	176	49	127	**0.001**^‡^	5	171	0.830^‡^	2	174	0.181^‡^
	Female	84	41	43		2	82		3	81
Location	Colon cancer	86	38	48	**0.000**^‡^	1	85	0.216^‡^	3	83	0.303^‡^
	Rectal cancer	140	52	88	0.294^‡^^¶^	6	134	0.188^‡^^¶^	1	139	0.125^‡^
	Gastric cancer	34	0	34		0	34		1	33
Histological type	Adenocarcinoma	230	79	151	**0.033**^‡^	7	223	0.919^‡^	5	225	0.964^‡^
	Mucinous adenocarcinoma	15	9	6		0	15		0	15	
	Low adhesion carcinoma	11	0	11		0	11		0	11	
	Signet-ring cell carcinoma	2	1	1		0	2		0	2	
	Squamous cell carcinoma	2	1	1		0	2		0	2	
Differentiation	Well	6	3	3	**0.036**^§^	0	6	0.506^§^	0	6	0.503^§^
	Moderate	182	69	113		6	176		3	179	
	Poor	72	18	54		1	71		2	70	
TNM stage	I	29	8	21	0.454^§^	0	29	0.585^§^	0	29	0.893^§^
	II	83	31	52		4	79		2	81	
	III	129	51	78		3	126		3	126	
	IV	19	0	19		0	19		0	19	
T	T1	4	1	3	0.500^§^	0	4	0.640^§^	0	4	0.824^§^
	T2	35	10	25		1	34		1	34	
	T3	195	70	125		6	189		3	192	
	T4	26	9	17		0	26		1	25	
N	Negative	111	39	72	0.879^‡^	4	107	0.433^‡^	2	109	0.902^‡^
	Positive	149	51	98		3	146		3	146	

† *t-test*.

‡ *Chi-square test*.

§ *Mann-Whitney test*.

¶ *Comparison between colon cancer and rectal cancer*.

*Bold values means P < 0.05, which indicate the statistically significantly difference*.

#### Colon cancer

**Figure 2:**
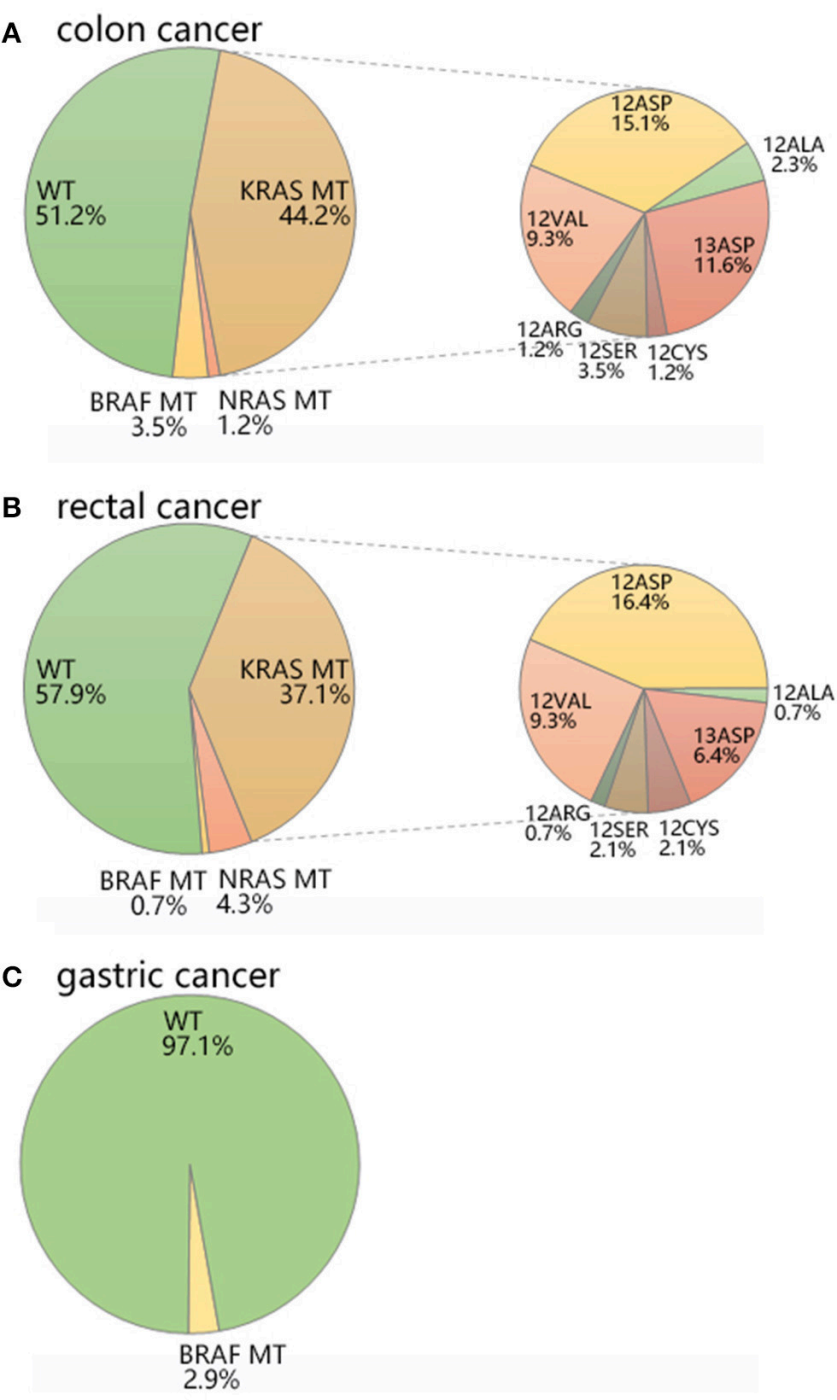
The mutation distribution of KRAS/NRAS/BRAF in **(A)** colon cancer, **(B)** rectal cancer, and **(C)** gastric cancer.

The average age of colon cancer patients was 63.53 ± 11.24 (Table 1). *KRAS*/*NRAS*/*BRAF* mutations were not significantly related to patients' age analyzed by Student's *t*-test or gender analyzed by Chi-square test in colon cancer. *KRAS* mutations were detected in 38 out of 86 (44.2%) colon cancer samples (Table 2, Figure 2A), of which 28 (73.7%) samples had mutations in codon 12 and 10 (26.3%) samples had mutations in codon 13 (Table 3). Among mutations in *KRAS* codon 12, the main mutant type was 12ASP (34.2%), followed by 12VAL (21.1%) (Table 3, Figure 2A). *KRAS* mutations occurred in all 7 sites included in this study. In contrast, *NRAS* had a lower mutation rate. *NRAS* mutations were detected in 1 out of 86 (1.2%) colon cancer samples (Table 2, Figure 2A). This mutation occurred in exon 3 codon 61 and the mutant type was Q61-Mu (Table 3). *BRAF* exon 15 codon 600 600Glu mutation was detected in 3 out of 86 (3.5%) colon cancer samples (Table 2, Figure 2A).

**Table 3:** Frequency and distribution of KRAS/NRAS/BRAF mutations.

**Gene**	**Exon**	**Codon**	**Mutation name**	**Amino acid**	**Nucleotide**	**COSMIC ID**	**Case**, ***n*** **(%)**			
							**Colon cancer**	**Rectal cancer**	**Gastric cancer**	**Total**
KRAS	2	12	12CYS	G12C	GGT>TGT	516	1 (2.6)	3 (5.7)	0 (0)	4 (4.4)
			12SER	G12S	GGT>AGT	517	3 (7.9)	3 (5.7)	0 (0)	6 (6.6)
			12ARG	G12R	GGT>CGT	518	1 (2.6)	1 (1.9)	0 (0)	2 (2.2)
			12VAL	G12V	GGT>GTT	520	8 (21.1)	13 (24.5)	0 (0)	21 (23.1)
			12ASP	G12D	GGT>GAT	521	13 (34.2)	23 (43.4)	0 (0)	36 (39.6)
			12ALA	G12A	GGT>GCT	522	2 (5.3)	1 (1.9)	0 (0)	3 (3.3)
		13	13ASP	G13D	GGC>GAC	532	10 (26.3)	9 (17.0)	0 (0)	19 (20.9)
NRAS	2	12	G12-Mu	G12D	GGT>GAT	564	0 (0)	3 (50.0)	0 (0)	3 (42.9)
		13	G13-Mu	G13R G13D G13V	GGT>CGT GGT>GAT GGT>GTT	569 573 574	0 (0)	2 (33.3)	0 (0)	2 (28.6)
	3	59	A59-Mu	A59D	GCT>GAT	253327	0 (0)	0 (0)	0 (0)	0 (0)
		61	Q61-Mu	Q61R	CAA>CGA	584	1 (100.0)	1 (1.67)	0 (0)	2 (28.6)
	4	117	K117-Mu	K117N K117N	AAG>AAC AAG>AAT	/ /	0 (0)	0 (0)	0 (0)	0 (0)
		146	A146-Mu	A146T	GCC>ACC	1237325	0 (0)	0 (0)	0 (0)	0 (0)
BRAF	15	600	600Glu	V600E	CTC>GAG	476	3 (100.0)	1 (100.0)	1 (100.0)	5 (100.0)

#### Rectal cancer

The average age of rectal cancer patients was 61.53 ± 10.39 (Table 1). *KRAS*/*NRAS*/*BRAF* mutations were not significantly related to patients' age analyzed by Student's *t*-test in rectal cancer. The mutation rate of *KRAS* gene in female (53.7%) is significantly higher than that of male (30.3%) (*p* = 0.012) analyzed by Chi-square test in rectal cancer. *KRAS* mutations were detected in 52 out of 140 (37.1%) rectal cancer samples (Table 2, Figure 2B), of which 43 (30.7%) samples had mutations in codon 12 and 9 (6.4%) samples had mutations in codon 13 (Table 3). Among mutations in *KRAS* codon 12, the main mutant type was 12ASP (16.4%), followed by 12VAL (9.3%) (Table 3, Figure 2B). *KRAS* mutations occurred in all 7 sites included in this study. It is worth noting that, there is one sample harbored both 12ASP and 12SER mutation. *NRAS* mutations were detected in 6 out of 140 (4.3%) rectal cancer samples (Table 2, Figure 2B), of which 3 (2.1%) samples had mutations in exon 2 codon 12, 2 (1.4%) samples had mutations in exon 2 codon 13, and 1 (0.7%) sample had mutations in exon 3 codon 61 (Table 3). None mutation was detected in *NRAS* exon 3 codon 59, exon 4 codon 117 and 146 in this study. *BRAF* exon 15 codon 600 600Glu mutation was detected in 1 out of 140 (0.7%) rectal cancer samples (Table 2, Figure 2B).

#### Gastric cancer

The average age of gastric cancer patients was 64.09 ± 8.74 (Table 1). *KRAS*/*NRAS*/*BRAF* mutations were not significantly related to patients' age analyzed by Student's *t*-test or gender analyzed by Chi-square test in gastric cancer. Compared with colon cancer and rectal cancer, *KRAS* and *NRAS* have a lower mutation rate in gastric cancer. In all 34 gastric cancer samples, neither *KRAS* nor *NRAS* mutation was detected, and only 1 sample (2.9%) was detected to have a mutation in *BRAF* exon 15 codon 600 600Glu (Table 2, Table 3, Figure 2C).

### Histopathological characteristics of mutations

#### Histological type

There were 5 histological types (adenocarcinoma, mucinous adenocarcinoma, low adhesion carcinoma, signet-ring cell carcinoma, squamous cell carcinoma) contained in the 260 investigated tumor samples. The mutation rate of *KRAS* was significantly different among the five histological types (*p* = 0.033). Among the five histological types, the mutation rates of *KRAS* were 34.3% in adenocarcinoma, 60.0% in mucinous adenocarcinoma, 0% in low adhesion carcinoma, 50% in signet-ring cell carcinoma, and 50% in squamous cell carcinoma, respectively. *NRAS* and *BRAF* mutations were only detected in 3.0 and 2.2% of adenocarcinomas, but not detected in other histological types. The mutation distribution is shown in Table 2.

#### Differentiation and staging

*KRAS* mutation rate was significantly different in different degrees of tissue differentiation (*p* = 0.036). The mutation rate was 50.0% in well differentiated cancers, 37.9% in moderate differentiated cancers, and 25.0% in poor differentiated cancers. There was no significant correlation between *KRAS*/*NRAS*/*BRAF* mutations and TNM stage, tumor infiltration depth, and lymph node metastasis. The mutation distribution is shown in Table 2.

#### IHC characteristics of mutations

IHC plays an important role in clinical pathology diagnosis. In the diagnostic process of CRC, BRAF (V600E), PMS2, EGFR, CDX2, CD34, Ki67, P53, MLH1, MSH6, and MSH2 are the most commonly used immunohistochemical markers for pathological typing, differential diagnosis of benign and malignant, and prognosis. Since only one sample was detected to have a mutation in BRAF in all 34 gastric cancer samples, we only investigated the IHC characteristics in colon cancers and rectal cancers.

Interestingly, mutations in the *BRAF* gene (1.77%) were not completely consistent with the IHC results of BRAF (V600E) (4.11%), but their correspondence is significant (*p* = 0.004). Moreover, there was a significant difference in the expression of EGFR between the *NRAS* MT and WT (*p* = 0.049); and there was a significant difference in the expression of MLH1 between the *BRAF* MT and WT (*p* = 0.004) (Table 4). When analyzing colon and rectal cancers separately, the results were similar. There was a significant difference in the expression of *BRAF* between the BRAF (V600E) MT and WT (*p* = 0.008) in colon cancers; and there was a significant difference in the expression of EGFR between the *NRAS* MT and WT (*p* = 0.021) in rectal cancers (Tables S1, S2). The representative IHC images for the markers were presented in Figure S1.

**Table 4:** Immunohistochemistry characteristics according to KRAS/NRAS/BRAF mutation status in CRC.

		**Total Case**	**KRAS (codon 12/13)**			**NRAS (codon 12/13/59/61/117/146)**			**BRAF (codon 600)**		
		**226**	**MT, *n***	**WT, *n***	***p*-value**	**MT, *n***	**WT, *n***	***p*-value**	**MT, *n***	**WT, *n***	***p*-value**
BRAF (V600E)	Positive	9	3	6	0.710^†^	0	9	1.000^‡^	2	7	**0.004**^‡^
	Negative	210	83	127		7	203		1	209	
	Missing	7	4	3		0	7		1	6	
PMS2	Positive	216	84	132	0.832^†^	7	209	1.000^‡^	2	214	0.092^‡^
	Negative	7	3	4		0	6		1	6	
	Missing	3	3	0		0	3		1	2	
EGFR	Positive	97	38	59	0.503^§^	5	92	**0.049**^§^	0	97	0.468^§^
	Weakly positive	74	26	48		1	73		3	71	
	Negative	48	23	25		0	48		0	48	
	Missing	7	3	4		1	6		1	6	
CDX2	Positive	219	86	133	0.663^†^	7	212	1.000^‡^	3	216	1.000^‡^
	Partially positive	4	2	2		0	4		0	4	
	Missing	3	2	1		0	3		0	3	
CD34	Positive	29	16	13	0.604^§^	0	29	0.800^§^	1	28	0.384^§^
	Vessel positive	42	12	30		3	39		1	41	
	Negative	92	37	55		2	90		1	91	
	Missing	63	25	38		2	61		1	62	
Ki67	Positive rate ≥90%	75	24	51	0.189^§^	4	71	0.257^§^	0	75	0.626^§^
	Positive rate 80~90%	69	29	40		1	68		2	67	
	Positive rate 70~80%	47	23	24		2	43		1	46	
	Positive rate 60~70%	23	9	14		0	23		0	23	
	Positive rate 50~60%	6	2	4		0	6		0	6	
	Positive rate <50%	3	1	2		0	3		0	3	
	Missing	3	2	1		0	3		1	2	
P53	Positive rate ≥90%	90	32	58	0.883^§^	4	86	0.540^§^	0	90	0.067^§^
	Positive rate 80~90%	17	10	7		0	17		0	17	
	Positive rate 70~80%	7	5	2		0	7		0	7	
	Positive rate 60~70%	3	1	2		0	3		0	3	
	Positive rate 50~60%	5	3	2		1	4		0	5	
	Positive rate <50%	36	14	22		0	36		2	34	
	Negative	64	22	42		2	62		1	63	
	Missing	4	3	1		0	4		1	3	
MLH1	Positive	193	74	119	0.431^§^	6	187	0.947^§^	1	192	**0.004**^§^
	Partially positive	23	8	15		1	22		1	22	
	Negative	6	5	1		0	6		1	5	
	Missing	4	3	1		0	4		1	3	
MSH6	Positive	193	74	119	0.511^§^	6	187	0.951^§^	3	190	0.501^§^
	Partially positive	22	10	12		1	21		0	22	
	Negative	7	3	4		0	7		0	7	
	Missing	4	3	1		0	4		1	3	
MSH2	Positive	202	80	122	0.655^§^	6	196	0.634^§^	2	200	0.103^§^
	Partially positive	17	7	10		1	16		0	17	
	Negative	3	0	3		0	3		1	2	
	Missing	4	3	1		0	4		1	3	

† *Chi-square test*.

‡ *Fisher's exact test*.

§ *Mann-Whitney test*.

*Bold values means P < 0.05, which indicate the statistically significantly difference*.

Of particular concern is the sample with double mutation sites on *KRAS*, and the immunohistochemistry results are as follows: BRAF (V600E) (–), PMS2 (+), EGFR (+), CDX2 (+), CD34 (–), Ki67 (positive rate 70%), P53 (–), MLH1 (+), MSH6 (+), MSH2 (+).

#### Correspondence between mutations and immunohistochemical markers

**Figure 3:**
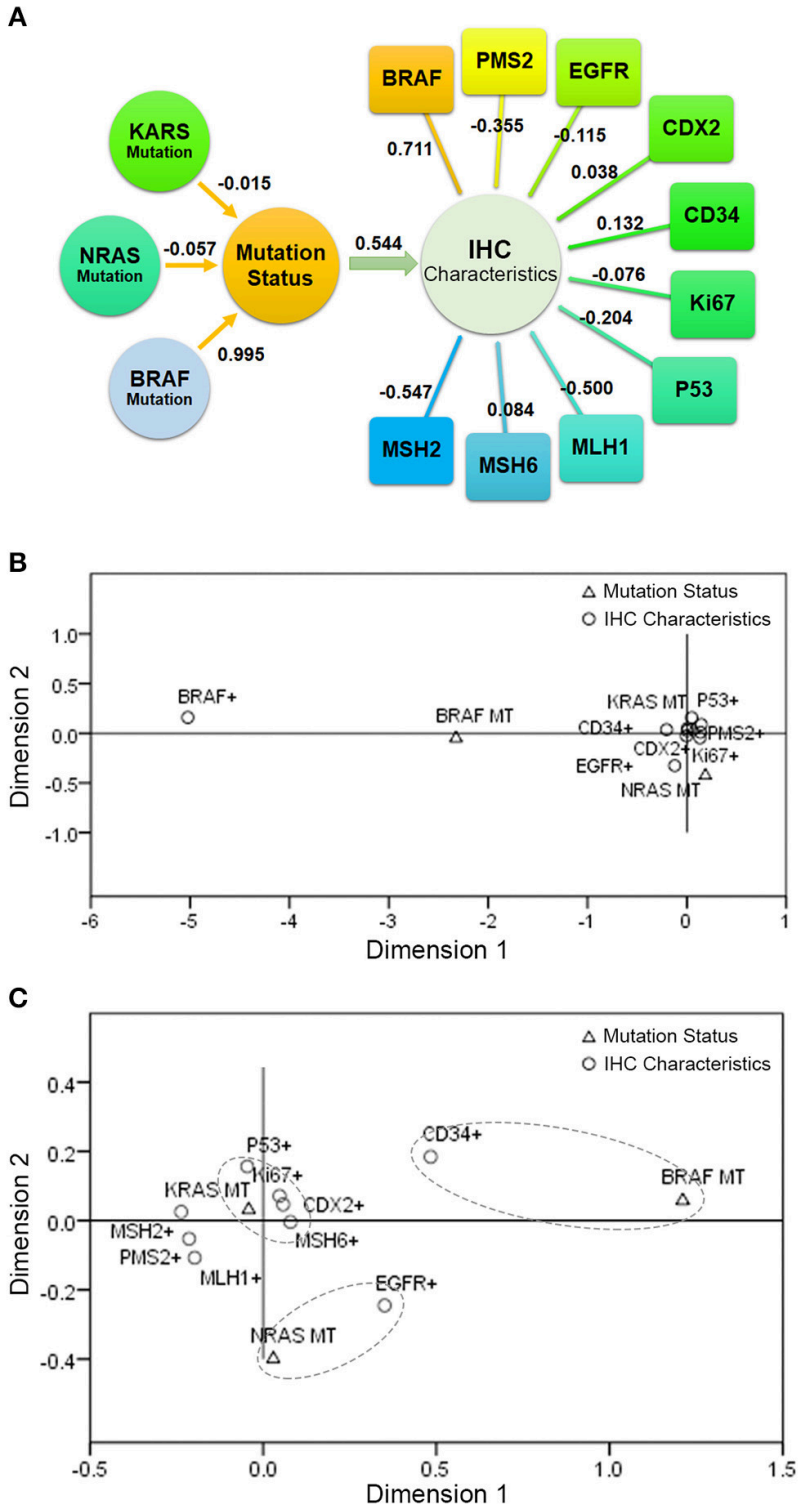
The correlation between KRAS/NRAS/BRAF mutation status and IHC characteristics in CRC. **(A)** The result of Canonical Correlation Analysis. **(B)** The result of Multiple Correspondence Analysis. **(C)** The result of Multiple Correspondence Analysis without the factor of BRAF (V600E) expression level.

In order to further explore the correlation between *KRAS*/*NRAS*/*BRAF* mutation status and IHC characteristics in CRC, Canonical Correlation Analysis and Multiple Correspondence Analysis were performed. The Canonical Correlation Analysis results showed that there is a strong correlation between mutation status and IHC characteristics (canonical correlation coefficient is 0.544). Among them, *BRAF* mutation status had a great influence on the mutation status (canonical correlation coefficient is 0.995); BRAF (V600E) expression level had a great influence on the IHC characteristics (canonical correlation coefficient is 0.711), followed by MSH2 (canonical correlation coefficient is −0.547) and MLH1 (canonical correlation coefficient is −0.500). The canonical correlation analysis structural diagram is shown in Figure 3A. The Multiple Correspondence Analysis results showed that there is a strong correspondence between BRAF mutation status and BRAF (V600E) expression level, which is theoretically obvious (Figure 3B). If the factor of BRAF (V600E) expression level is excluded and re-analyzed, the results showed that the *KRAS* mutation status had a certain relationship with the expression of P53, Ki67, CDX2, and MSH6; the *NRAS* mutation status had a certain relationship with the expression of EGFR; and the *BRAF* mutation status had a certain relationship with the expression of CD34 (Figure 3C).

### Correspondence between mutations and prognosis

**Figure 4:**
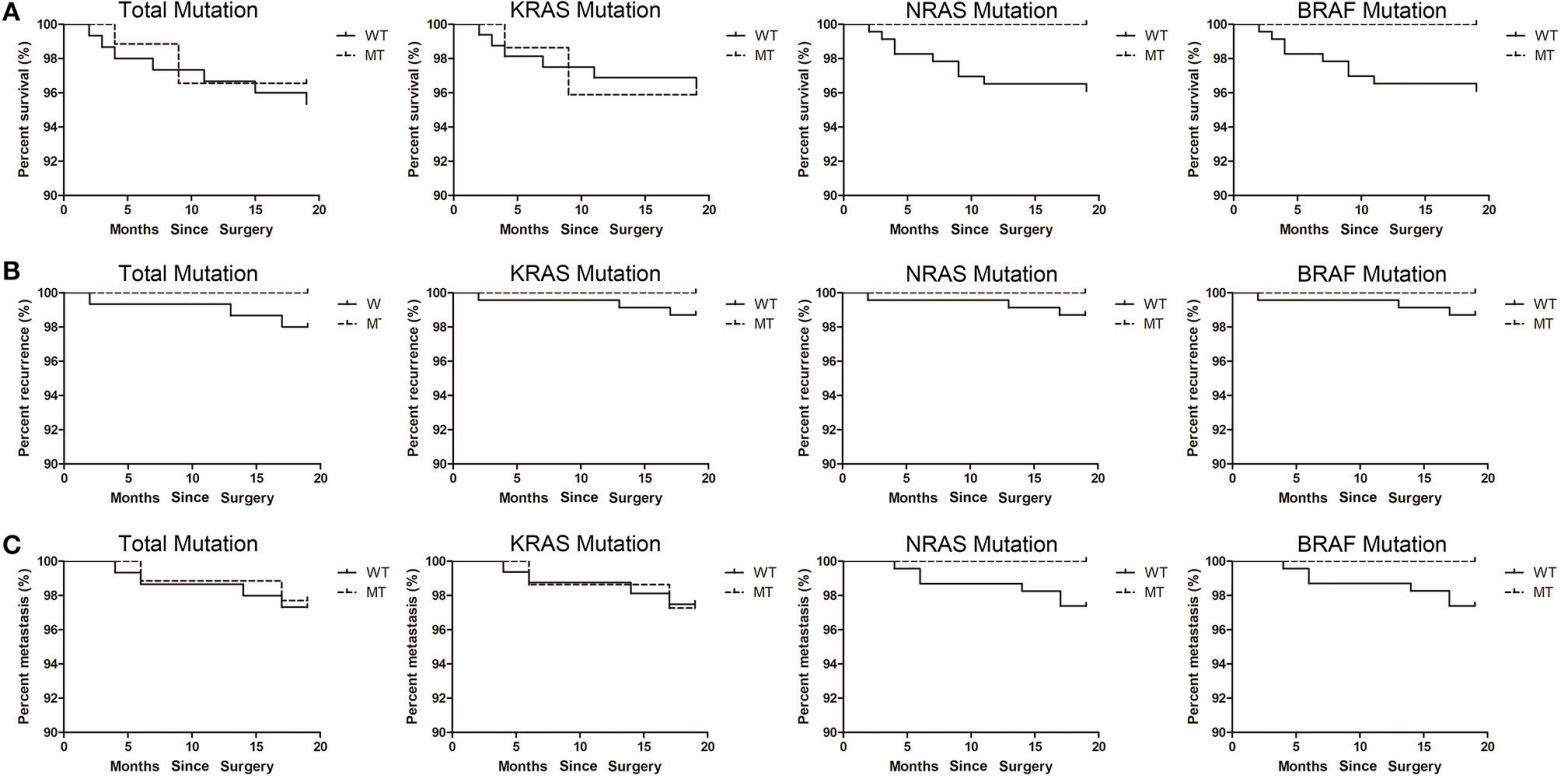
The correspondence between mutations and prognosis. **(A)** The correspondence between mutations and survival. **(B)** The correspondence between mutations and recurrence. **(C)** The correspondence between mutations and metastasis.

Since the sampling period of this study is only 19 months, it is difficult to accurately reflect the correspondence between gene mutation and prognosis. Therefore, we only summarized the prognosis information of patients up to the present stage. According to the type of mutation, the correspondences between mutation and survival, recurrence and metastasis were analyzed separately. No significant difference was found between mutant type and wild type patients. The correspondence between mutations and survival was shown in Figure 4A, the correspondence between mutations and recurrence was shown in Figure 4B, the correspondence between mutations and metastasis was shown in Figure 4C.

## Discussion

In this study, we investigated the mutation status of *KRAS* (exons 2, codon 12/13), *NRAS* (exons 2/3/4, codon 12/13/59/61/117/146) and *BRAF* (exons 15, codon 600) in 260 patients, including 86 cases of colon cancer, 140 cases of rectal cancer and 34 cases of gastric cancer. The results showed that *KRAS* mutations were detected in 44.2% colon cancer, 37.1% rectal cancer and none in gastric cancer; *NRAS* mutations were detected in 1.2% colon cancer, 4.3% rectal cancer and none in gastric cancer; *BRAF* mutations were detected in 3.5% colon cancer, 0.7% rectal cancer, and 2.9% in gastric cancer.

The mutation rate of *KRAS* gene in female (48.8%) is significantly higher than that of male (27.8%). *KRAS* gene mutation rate was significantly different in colon cancers (44.2%), rectal cancers (37.1%) and gastric cancers (0%), however, when colon cancer and rectal cancer were compared alone, the difference was not significant.

Compared with the previous studies (Table 5), the mutation rate of *KRAS* in CRC reported in our study was consistent with that reported by Douillard et al. (6) and Gao et al. (25), who also focused on exons 2, codon 12/13. Guo et al. (13) believed that the mutation rate of *KRAS* was as high as 52.7%. This conclusion may be because they included more genetic loci into the study (exons 2/3/4, codons 12/13/59/61/117/146/147). Compared to the mutation rate of KRAS, there are fewer studies focusing on the mutation rate of *NRAS*. The mutation rate of *NRAS* in CRC obtained in this study was in the midstream position compared to other reports (4, 6, 13, 14, 16). Vaughn et al showed that the mutation rate of *NRAS* in Americans was only 1.2% (24). We measured a low mutation rate of *BRAF* in CRC. This result is consistent with the research on Chinese carried by Shen et al. (27). In the study of Mao et al. (20), the *BRAF* mutation rate reached a staggering 25.4%, much higher than other reports. However, the sample size in that study was only 69 cases, and its representativeness was questionable. By comparison, it can be found that the mutation rate of these three genes is not significantly different between Asians and Europeans.

**Table 5:** Studies on mutation status of KRAS, NRAS and BRAF in CRC.

**Reference (year)**	**Sample size**	**Method**	**Mutation frequencies**			**Region**
			**KRAS**	**NRAS**	**BRAF**	
This study	226	ARMS-PCR	39.82% (exons 2, codon 12/13)	3.10% (exons 2/3/4, codon 12/13/59/61/117/146)	1.77% (exons 15, codon 600)	China
Guo F, et al. (13)	353	Direct sequencing	52.7% (exons 2/3/4, codons 12/13/59/61/117/146/147)	3.4% (exons 2/3, codons 12/13/60/61)	4.5% (exons 15, codons 600/601)	China
Zhang et al. (14)	1110	ARMS-PCR	45.4% (exons 2/3/4, codons 12/13/61/117/146)	3.9% (exons 2/3/4, codon 12/13/61/146)	3.1% (exon 15, codon 600)	China
Tong et al. (15)	1506	Direct sequencing	44.5% (exons 2/3/4, codons 12/13/61/146)			Hong Kong, China
Douillard et al. (6)	1183	Direct sequencing	40.1% (exons 2, codons 12/13)	4.1% (exons 2/3/4, codon 12/13/61/117/146)	4.5% (exon 15, codon 600)	France
Shen et al. (16)	676	Direct sequencing	35.9% (exons 2/3, codons 12/13/61)	4.19% (exons 2/3, codons 12/13/61)	6.96% (exon 11/15)	China
Pu et al. (17)	115	Direct sequencing	32.2% (exons 2, codons 12/13)		3.5% (exons 15, codon 600)	China
Wang et al. (18)	574	Direct sequencing	34.2% (exons 2/3, codons 12/13/61)			China
Chang et al. (19)	165	High-resolution melting	36.97% (exons 2/3, codons 12/13/61		4.24% (exons 15, codon 600)	Taiwan, China
Mao et al. (20)	69	Direct sequencing	43.9% (exons 2, codons 12/13/14)		25.4% (exons 15, codon 600)	China
Hsieh et al. (21)	182	Direct sequencing & high-resolution melting	33.5% (exons 2, codons 12/13)		1.1% (exons 15, codon 600)	Taiwan, China
Li et al. (22)	78	Direct sequencing	33.3% (exons 2, codons 12/13)			China
Yokota et al. (23)	229	Cycleave PCR	34.5% (exons 2/3, codons 12/13/61)		6.5% (exon 15, codon 600)	Japan
Vaughn et al. (24)	2121	Pyrosequencing	42.4% (exons 2, codons 12/13)	1.2% (exons 2/3, codons 12/13/61)	3.7% (exon 15, codon 600)	US
Gao et al. (25)	273	Direct sequencing	38.5% (exons 2, codon 12/13)		5.1% (exon 15, codon 600)	China
Li et al. (26)	200	Pyrosequencing	31.5% (exon 2, codon 12/13)		7.0% (exons 15, codon 600)	China
Shen et al. (27)	118	Pyrosequencing	34.7% (exons 2/3, codons 12/13/61)		1.7% (exons 15, codon 600)	China
Liou et al. (28)	314	Direct sequencing	20.7% (exons 2/3, codons 12/13/61)		3.8% (exon 11/15)	Taiwan, China
De Roock et al. (4)	1022	Mass spectrometry genotyping	40.0% (exons 2/3, codons 12/13/61/146)	2.6% (exons 2/3, codons 12/13/61)	4.7% (exon 15, codon 594/600)	seven European countries

There are many reasons for the different mutation rate results. In addition to the influence of the sample size, different mutation sites included in the study will result in different mutation rates. *KRAS* mutations occur 98.5% in exon 2 at codons 12 and 13. The common mutation site of *NRAS* gene is located in exons 2, 3, and 4. About 81.9% *BRAF* mutations are located at codon 600. Therefore, in this study we focused our attention on the mutations at these sites. Another factor that may affect the outcome of the mutation rate is the detection method. Direct sequencing is the most widely used method for mutation detection (15, 17, 18, 22, 28). It is the gold standard for mutation detection, but it is limited by sensitivity, and only mutations with a mutational heterogeneity more than 10% can be detected. Besides, pyrosequencing (24, 26, 27), high-resolution melting (19, 21), ARMS-PCR(14), cycleave PCR(23) and mass spectrometry genotyping (4) are also used for the detection of mutations. In this study we applied two-color fluorescent probe ARMS-PCR. This method is more sensitive than direct sequencing, as little as 1% of heterogeneous mutations in tumor tissue can be detected. The type of sample to be tested may also have a certain effect on the mutation rate results. In this study, fresh tissue without being fixed by paraformaldehyde was used to avoid DNA damage during the fixation process.

The mutation rate of *KRAS* was significantly different in different histological types, *NRAS* and *BRAF* mutations were only detected in adenocarcinomas. Furthermore, we found that *KRAS* gene mutation rate was significantly different in different degrees of tissue differentiation, but not significantly associated with TNM stage. *KRAS* mutation rate increased with the higher degree of differentiation. These results were a little different from those reported by Guo et al. (13), who termed *KRAS* mutations had no significant correlation with clinicopathological characteristics.

None of the previous studies investigated the association between *KRAS*/*NRAS*/*BRAF* mutations and IHC characteristics in CRCs. In our study, we investigated the association between *KRAS*/*NRAS*/*BRAF* mutations and commonly used immunohistochemical markers, including BRAF (V600E), PMS2, EGFR, CDX2, CD34, Ki67, P53, MLH1, MSH6, and MSH2. We were surprised to find that the mutations in *BRAF* gene were not completely consistent with the IHC results of BRAF (V600E), the mutation rate of *BRAF* detected by ARMS-PCR (1.77%) was significantly lower than that by IHC (4.11%). Molecular testing is the gold standard for genetic mutation detection. Although many studies have shown that IHC has a good detection effect on BRAF V600E mutations (29–32), but there will still be a part of false positive results (33, 34). Ballester et al. (35) suggested that the highly sensitive molecular assays remain the gold standard for BRAF mutation analysis in paraffin-embedded lesions. Ehsani et al. (36) used IHC to detect BRAF mutations in metastatic malignant melanoma with a false positive rate of 32%. We suggest that if the purpose of detecting *BRAF* mutations is to guide anti-*EGFR* targeted therapy, genetic testing will benefit more patients rather than IHC. Moreover, we also found that there was a significant difference in the expression of EGFR between the *NRAS* MT and WT; and there was a significant difference in the expression of MLH1 between the *BRAF* MT and WT. Parsons et al. (37) reported that tumor *BRAF* mutation, and MLH1 promoter “C region” methylation specifically, are strong predictors of negative MMR mutation status in CRCs. Farchoukh et al. (38) also found that although the presence of the BRAF mutation is indicative of a sporadic cancer, up to 30–50% of colorectal carcinomas with MLH1 promoter hypermethylation will lack a *BRAF* mutation. Some similar studies have also shown that *BRAF* mutation is closely linked with MLH1 promoter hypermethylation and DNA mismatch repair (MMR) gene mutations, but its specific mechanism needs further study (39).

Furthermore, we employed Canonical Correlation Analysis and Multiple Correspondence Analysis to further explore the correlation between *KRAS*/*NRAS*/*BRAF* mutation status and IHC characteristics in CRC. The results indicated that the *KRAS* mutation status had a certain relationship with the expression of P53, Ki67, CDX2, and MSH6; the *NRAS* mutation status had a certain relationship with the expression of EGFR; and the *BRAF* mutation status had a certain relationship with the expression of CD34. There are few direct reports of correlation studies between these genes. We hypothesize that since *KRAS, NRAS*, and *BRAF* are potential tumor-driven genes, their mutation may have some synergistic or inhibitory effects on the expression of genes such as P53, Ki67, CDX2, MSH6, and CD34.

There are few studies on the mutations rate of *KRAS*/*NRAS*/*BRAF* in gastric cancer. And gastric cancer patients benefit little from anti-*EGFR* MoAbs targeted therapy. Compared with colon cancer rectal cancer, *KRAS*/*NRAS*/*BRAF* have a lower mutation rate in gastric cancer, furthermore, there is no consistent conclusion on the role of *KRAS*/*NRAS*/*BRAF* mutations in gastric cancer (40–43). In this study we found 1 out of 34 gastric cancer cases with *BRAF* mutation. No *KRAS* or *NRAS* mutation was found in gastric cancer in this study. Here we provide these data for further research by peers.

Since the sampling period of this study is between November 2016 and June 2018, it is difficult to accurately reflect the correspondence between gene mutation and prognosis. Therefore, we only summarized the prognosis information of patients up to the present stage. We will continue to follow this group of patients in subsequent studies to delve into the effects of *KRAS*/*NRAS*/*BRAF* mutation on prognosis.

The limitations of our study are its relatively small sample size, and lack of follow-up time which are important for risk assessment of malignant tumor. *NRAS* and *BRAF* mutation frequency was too low to analyze its mutation subgroups. The specific mechanism and clinical significance of the relationship between *KRAS*/*NRAS*/*BRAF* mutation and IHC status still need further experiments to confirm.

In this study, we systematically described and statistically analyzed the frequencies and distributions of *KRAS*/*NRAS*/*BRAF* genetic mutation status and their relationship with IHC in 260 cases with colorectal cancer or gastric cancer through retrospective analysis. Based on the analysis results, we draw the following conclusions: (1) *KRAS* mutations were detected in 44.2% colon cancer, 37.1% rectal cancer and none in gastric cancer; *NRAS* mutations were detected in 1.2% colon cancer, 4.3% rectal cancer and none in gastric cancer; *BRAF* mutations were detected in 3.5% colon cancer, 0.7% rectal cancer and 2.9% in gastric cancer; (2) the mutation rate of *KRAS* in female (48.8%) was significantly higher than that of male (27.8%); (3) the mutation rate increased with the higher degree of differentiation; (4) the mutation rate of *BRAF* detected by ARMS-PCR (1.77%) was significantly lower than that by IHC (4.11%); (5) the *KRAS*/*NRAS*/*BRAF* mutation status had a certain relationship with the expression of some common immunohistochemical markers. This study provides more data support for clinical research on *KRAS*/*NRAS*/*BRAF* mutation in CRCs or gastric cancers. In the context of precision medicine, more precise classification of genetic profile should be implemented to enhance the clinical experience. Our study suggested that, combining genetic mutations with immunohistochemical phenotypes could help doctors to formulate cancer treatment strategies more accurately with combining chemotherapy, immunotherapy, and targeted therapy. However, the specific mechanism and clinical significance of the relationship between *KRAS*/*NRAS*/*BRAF* mutation and IHC status still need further experiments to confirm.

## Author contributions

QY conducted experimental operations, sample processing, data analysis, and article writing. YS and HZ conducted experimental operations, sample processing, and data analysis. SH, YL, WL and XW performed sample pretreatment. ZD carried out experimental design and sample pretreatment. TL and GZ conceived and designed the experiments.

### Conflict of interest statement

The authors declare that the research was conducted in the absence of any commercial or financial relationships that could be construed as a potential conflict of interest.

## Supplementary material

Figure S1 The representative HE and IHC images for the markers.

Table S1 Immunohistochemistry characteristics according to KRAS/NRAS/BRAF gene mutation status in colon cancer.

Table S2 Immunohistochemistry characteristics according to KRAS/NRAS/BRAF gene mutation status in rectal cancer.

## References

[1] Siegel RL Miller KD Jemal A. Cancer statistics, 2018. CA Cancer J Clin. (2018) 68:7–30. 10.3322/caac.21442 29313949

[2] Siegel RL Miller KD Fedewa SA Ahnen DJ Meester RGS Barzi A. Colorectal cancer statistics, 2017. CA Cancer J Clin. (2017) 67:177–93. 10.3322/caac.21395 28248415

[3] Van Cutsem E Cervantes A Adam R Sobrero A Van Krieken JH Aderka D. ESMO consensus guidelines for the management of patients with metastatic colorectal cancer. Ann Oncol. (2016) 27:1386–422. 10.1093/annonc/mdw235 27380959

[4] De Roock W Claes B Bernasconi D De Schutter J Biesmans B Fountzilas G. Effects of <em>KRAS, BRAF, NRAS</em>, and <em>PIK3CA</em> mutations on the efficacy of cetuximab plus chemotherapy in chemotherapy-refractory metastatic colorectal cancer: a retrospective consortium analysis. Lancet Oncol. (2010) 11:753–62. 10.1016/S1470-2045(10)70130-3 20619739

[5] Li K Guo Q Yang J Chen H Hu K Zhao J. FOXD3 is a tumor suppressor of colon cancer by inhibiting EGFR-Ras-Raf-MEK-ERK signal pathway. Oncotarget (2017) 8:5048–56. 10.18632/oncotarget.13790 27926503 PMC5354891

[6] Douillard JY Oliner KS Siena S Tabernero J Burkes R Barugel M. Panitumumab-FOLFOX4 treatment and RAS mutations in colorectal cancer. N Engl J Med. (2013) 369:1023–34. 10.1056/NEJMoa1305275 24024839

[7] Popovici V Budinska E Bosman FT Tejpar S Roth AD Delorenzi M. Context-dependent interpretation of the prognostic value of BRAF and KRAS mutations in colorectal cancer. BMC Cancer (2013) 13:439. 10.1186/1471-2407-13-439 24073892 PMC3849526

[8] Dienstmann R Salazar R Tabernero J. Personalizing colon cancer adjuvant therapy: selecting optimal treatments for individual patients. J Clin Oncol. (2015) 33:1787–96. 10.1200/jco.2014.60.0213 25918287

[9] Schmoll HJ Van Cutsem E Stein A Valentini V Glimelius B Haustermans K. ESMO consensus guidelines for management of patients with colon and rectal cancer. a personalized approach to clinical decision making. Ann Oncol. (2012) 23:2479–516. 10.1093/annonc/mds236 23012255

[10] Baas JM Krens LL Guchelaar HJ Morreau H Gelderblom H. Concordance of predictive markers for EGFR inhibitors in primary tumors and metastases in colorectal cancer: a review. Oncologist (2011) 16:1239–49. 10.1634/theoncologist.2011-0024 21742964 PMC3228174

[11] Benson AB 3rd Venook AP Cederquist L Chan E Chen YJ Cooper HS. Colon Cancer, Version 1.2017, NCCN clinical practice guidelines in oncology. J Natl Compr Canc Netw. (2017) 15:370–98. 28275037 10.6004/jnccn.2017.0036

[12] Er TK Chen CC Bujanda L Herreros-Villanueva M. Clinical relevance of KRAS mutations in codon 13: where are we? Cancer Lett. (2014) 343:1–5. 10.1016/j.canlet.2013.09.012 24051306

[13] Guo F Gong H Zhao H Chen J Zhang Y Zhang L. Mutation status and prognostic values of KRAS, NRAS, BRAF and PIK3CA in 353 Chinese colorectal cancer patients. Sci Rep. (2018) 8:6076. 10.1038/s41598-018-24306-1 29666387 PMC5904111

[14] Zhang J Zheng J Yang Y Lu J Gao J Lu T. Molecular spectrum of KRAS, NRAS, BRAF and PIK3CA mutations in Chinese colorectal cancer patients: analysis of 1,110 cases. Sci Rep. (2015) 5:18678. 10.1038/srep18678 26691448 PMC4687048

[15] Tong JH Lung RW Sin FM Law PP Kang W Chan AW. Characterization of rare transforming KRAS mutations in sporadic colorectal cancer. Cancer Biol Ther. (2014) 15:768–76. 10.4161/cbt.28550 24642870 PMC4049792

[16] Shen Y Wang J Han X Yang H Wang S Lin D. Effectors of epidermal growth factor receptor pathway: the genetic profiling ofKRAS, BRAF, PIK3CA, NRAS mutations in colorectal cancer characteristics and personalized medicine. PLoS ONE (2013) 8:e81628. 10.1371/journal.pone.0081628 24339949 PMC3858242

[17] Pu X Pan Z Huang Y Tian Y Guo H Wu L. Comparison of KRAS/BRAF mutations between primary tumors and serum in colorectal cancer: Biological and clinical implications. Oncol Lett. (2013) 5:249–54. 10.3892/ol.2012.963 23255930 PMC3525490

[18] Wang J Yang H Shen Y Wang S Lin D Ma L. Direct sequencing is a reliable assay with good clinical applicability for KRAS mutation testing in colorectal cancer. Cancer Biomark. (2013) 13:89–97. 10.3233/cbm-130334 23838137 PMC12928280

[19] Chang YS Chang SJ Yeh KT Lin TH Chang JG. RAS, BRAF, and TP53 gene mutations in Taiwanese colorectal cancer patients. Onkologie (2013) 36:719–24. 10.1159/000356814 24356563

[20] Mao C Zhou J Yang Z Huang Y Wu X Shen H. KRAS, BRAF and PIK3CA mutations and the loss of PTEN expression in Chinese patients with colorectal cancer. PLoS ONE (2012) 7:e36653. 10.1371/journal.pone.0036653 22586484 PMC3346734

[21] Hsieh LL Er TK Chen CC Hsieh JS Chang JG Liu TC. Characteristics and prevalence of KRAS, BRAF, and PIK3CA mutations in colorectal cancer by high-resolution melting analysis in Taiwanese population. Clin Chim Acta (2012) 413:1605–11. 10.1016/j.cca.2012.04.029 22579930

[22] Li Z Chen Y Wang D Wang G He L Suo J. Detection of KRAS mutations and their associations with clinicopathological features and survival in Chinese colorectal cancer patients. J Int Medical Res. (2012) 40:1589–98. 10.1177/147323001204000439 22971512

[23] Yokota T Ura T Shibata N Takahari D Shitara K Nomura M. BRAF mutation is a powerful prognostic factor in advanced and recurrent colorectal cancer. Br J Cancer (2011) 104:856–62. 10.1038/bjc.2011.19 21285991 PMC3048210

[24] Vaughn CP Zobell SD Furtado LV Baker CL Samowitz WS. Frequency of KRAS, BRAF, and NRAS mutations in colorectal cancer. Genes Chromosomes Cancer (2011) 50:307–12. 10.1002/gcc.20854 21305640

[25] Gao J Wang TT Yu JW Li YY Shen L. Wild-Type KRAS and BRAF could predict response to cetuximab in chinese colorectal cancer patients. Chin J Cancer Res. (2011) 23:271–5. 10.1007/s11670-011-0271-4 23357879 PMC3551306

[26] Li HT Lu YY An YX Wang X Zhao QC. KRAS, BRAF and PIK3CA mutations in human colorectal cancer: relationship with metastatic colorectal cancer. Oncol. Rep. (2011) 25:1691–7. 10.3892/or.2011.1217 21424126

[27] Shen H Yuan Y Hu HG Zhong X Ye XX Li MD. Clinical significance of K-ras and BRAF mutations in Chinese colorectal cancer patients. World J Gastroenterol. (2011) 17:809–16. 10.3748/wjg.v17.i6.809 21390154 PMC3042662

[28] Liou JM Wu MS Shun CT Chiu HM Chen MJ Chen CC. Mutations in BRAF correlate with poor survival of colorectal cancers in Chinese population. Int J Colorectal Dis. (2011) 26:1387–95. 10.1007/s00384-011-1229-1 21553007

[29] Kim JK Seong CY Bae IE Yi JW Yu HW Kim SJ. Comparison of immunohistochemistry and direct sequencing methods for identification of the BRAF(V600E) mutation in papillary thyroid carcinoma. Ann Surgical Oncol. (2018) 25:1775–81. 10.1245/s10434-018-6460-3 29611028

[30] Fisher KE Cohen C Siddiqui MT Palma JF Lipford EH 3rd Longshore JW. Accurate detection of BRAF p.V600E mutations in challenging melanoma specimens requires stringent immunohistochemistry scoring criteria or sensitive molecular assays. Hum Pathol. (2014) 45:2281–93. 10.1016/j.humpath.2014.07.014 25228337

[31] Ilie MI Lassalle S Long-Mira E Bonnetaud C Bordone O Lespinet V. Diagnostic value of immunohistochemistry for the detection of the BRAF(V600E) mutation in papillary thyroid carcinoma: comparative analysis with three DNA-based assays. Thyroid (2014) 24:858–66. 10.1089/thy.2013.0302 24417277

[32] Long GV Wilmott JS Capper D Preusser M Zhang YE Thompson JF. Immunohistochemistry is highly sensitive and specific for the detection of V600E BRAF mutation in melanoma. Am J Surg Pathol. (2013) 37:61–5. 10.1097/PAS.0b013e31826485c0 23026937

[33] Jabbar KJ Luthra R Patel KP Singh RR Goswami R Aldape KD. Comparison of next-generation sequencing mutation profiling with BRAF and IDH1 mutation-specific immunohistochemistry. Am J Surg Pathol (2015) 39:454–61. 10.1097/pas.0000000000000325 25634750

[34] Zimmermann AK Camenisch U Rechsteiner MP Bode-Lesniewska B Rossle M. Value of immunohistochemistry in the detection of BRAF(V600E) mutations in fine-needle aspiration biopsies of papillary thyroid carcinoma. Cancer Cytopathol. (2014) 122:48–58. 10.1002/cncy.21352 24039206

[35] Ballester LY Cantu MD Lim KPH Sarabia SF Ferguson LS Renee Webb C. The use of BRAF V600E mutation-specific immunohistochemistry in pediatric Langerhans cell histiocytosis. Hematol Oncol. (2018) 36:307–15. 10.1002/hon.2388 28219109 PMC6886693

[36] Ehsani L Cohen C Fisher KE Siddiqui MT. BRAF mutations in metastatic malignant melanoma: comparison of molecular analysis and immunohistochemical expression. Appl Immunohistochem Mole Morphol. (2014) 22:648–51. 10.1097/pai.0000000000000013 25046227

[37] Parsons MT Buchanan DD Thompson B Young JP Spurdle AB. Correlation of tumour BRAF mutations and MLH1 methylation with germline mismatch repair (MMR) gene mutation status: a literature review assessing utility of tumour features for MMR variant classification. J Med Genet. (2012) 49:151–7. 10.1136/jmedgenet-2011-100714 22368298

[38] Farchoukh L Kuan SF Dudley B Brand R Nikiforova M Pai RK. MLH1-deficient colorectal carcinoma with wild-type BRAF and MLH1 promoter hypermethylation harbor KRAS mutations and arise from conventional adenomas. Am J Surg Pathol. (2016) 40:1390–9. 10.1097/pas.0000000000000695 27438990

[39] Adar T Rodgers LH Shannon KM Yoshida M Ma T Mattia A. A tailored approach to BRAF and MLH1 methylation testing in a universal screening program for Lynch syndrome. Mod Pathol. (2017) 30:440–7. 10.1038/modpathol.2016.211 28059100

[40] Lee SH Lee JW Soung YH Kim HS Park WS Kim SY. BRAF and KRAS mutations in stomach cancer. Oncogene (2003) 22:6942–5. 10.1038/sj.onc.1206749 14534542

[41] Takahashi N Yamada Y Taniguchi H Fukahori M Sasaki Y Shoji H. Clinicopathological features and prognostic roles of KRAS, BRAF, PIK3CA and NRAS mutations in advanced gastric cancer. BMC Res. Notes (2014) 7:271. 10.1186/1756-0500-7-271 24774510 PMC4012089

[42] Lee SH Ahn BK Baek SU Chang HK. BRAF mutation in multiple primary cancer with colorectal cancer and stomach cancer. Gastroenterol Rep. (2013) 1:70–4. 10.1093/gastro/got004 24759670 PMC3941443

[43] Lu W Wei H Li M Wang H Liu L Zhang Q. Identification of KRAS and PIK3CA but not BRAF mutations in patients with gastric cancer. Mole Med Rep. (2015) 12:1219–24. 10.3892/mmr.2015.3530 25815786

