# Supplementary figures and images for: Mutation Status and Immunohistochemical Correlation of KRAS, NRAS, and BRAF in 260 Chinese Colorectal and Gastric Cancers
Source: Front Oncol. 2018 Oct 26;8:487. doi: 10.3389/fonc.2018.00487 (PMC6212577; doi:10.3389/fonc.2018.00487)

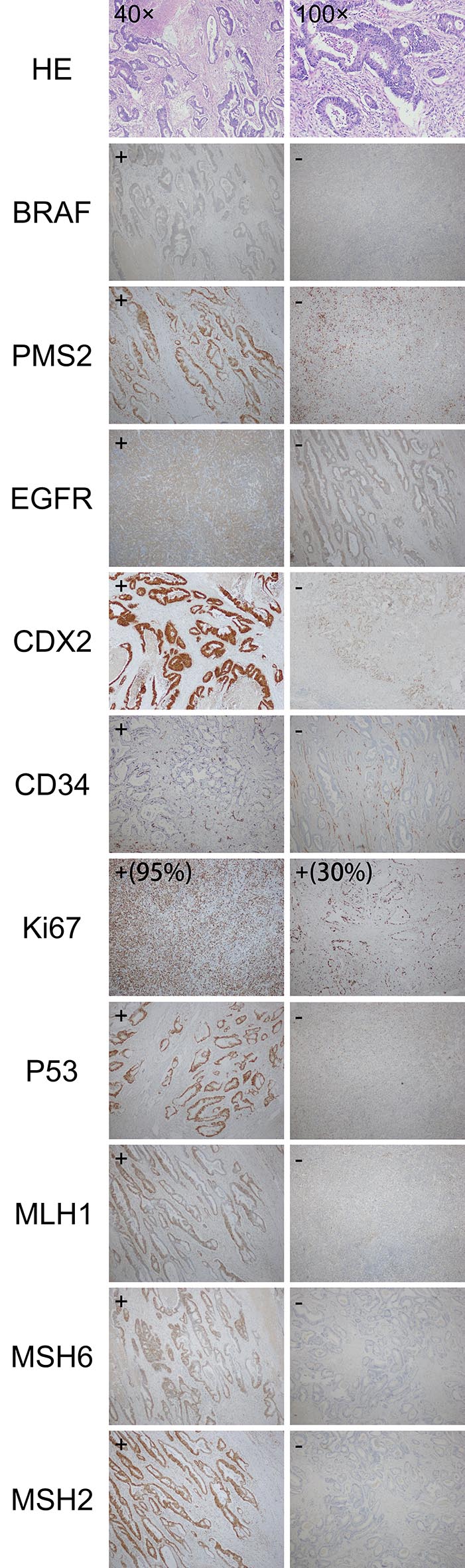

Supplement: Figure S1 — The representative HE and IHC images for the markers. [file Image_1.JPEG]
