# Supplementary material for: Mutation Status and Immunohistochemical Correlation of KRAS, NRAS, and BRAF in 260 Chinese Colorectal and Gastric Cancers
Source: Front Oncol. 2018 Oct 26;8:487. doi: 10.3389/fonc.2018.00487 (PMC6212577; doi:10.3389/fonc.2018.00487)
Supplement: Table S1 — Immunohistochemistry characteristics according to KRAS/NRAS/BRAF gene mutation status in colon cancer. [file Table_1.DOCX]

Supplementary Material

**Mutation status and immunohistochemical correlation of *KRAS*, *NRAS* and *BRAF* in 260 Chinese colorectal and gastric cancers**

Qiwei Yang^1^, Sibo Huo^2^, Yujie Sui^1^, Zhenwu Du^1,3^, Haiyue Zhao^4^, Yu Liu^2^, Wei Li^2^, Xin Wan^2^, Tongjun Liu^2*^, Guizhen Zhang^1,3*^

***Correspondence:** Professor. Guizhen Zhang: [zhangguizhenjlu@163.com](mailto:zhangguizhenjlu@163.com) & Professor. Tongjun Liu [tongjunliu@163.com](mailto:tongjunliu@163.com)

Table S1. Immunohistochemistry characteristics according to KRAS/NRAS/BRAF gene mutation status in colon cancer.

|  |  | Total Case | KRAS (codon 12/13) | | | NRAS (codon 12/13/59/61/117/146) | | | BRAF (codon 600) | | |
| --- | --- | --- | --- | --- | --- | --- | --- | --- | --- | --- | --- |
|  |  | 86 | MT, n | WT, n | p vale | MT, n | WT, n | p vale | MT, n | WT, n | p vale |
| BRAF (V600E) | Positive | 5 | 2 | 3 | 0.870 ^†^ | 0 | 5 | 1.000 ^‡^ | 2 | 3 | **0.008** ^‡^ |
|  | Negative | 80 | 35 | 45 |  | 1 | 79 |  | 1 | 79 |  |
|  | Missing | 1 | 1 | 0 |  | 0 | 1 |  | 0 | 1 |  |
| PMS2 | Positive | 80 | 35 | 45 | 0.870 ^†^ | 1 | 79 | 1.000 ^‡^ | 2 | 78 | 0.168 ^‡^ |
|  | Negative | 5 | 2 | 3 |  | 0 | 5 |  | 1 | 4 |  |
|  | Missing | 1 | 1 | 0 |  | 0 | 1 |  | 0 | 1 |  |
| EGFR | Positive | 35 | 18 | 17 | 0.557 ^§^ | 0 | 35 | 0.771 ^§^ | 0 | 35 | 0.507 ^§^ |
|  | Weakly positive | 32 | 11 | 21 |  | 1 | 31 |  | 3 | 29 |  |
|  | Negative | 16 | 8 | 8 |  | 0 | 16 |  | 0 | 16 |  |
|  | Missing | 3 | 1 | 2 |  | 0 | 3 |  | 0 | 3 |  |
| CDX2 | Positive | 84 | 36 | 48 | 0.192 ^‡^ | 1 | 83 | 1.000 ^‡^ | 3 | 81 | 1.000 ^‡^ |
|  | Partially positive | 2 | 2 | 0 |  | 0 | 2 |  | 0 | 2 |  |
|  | Missing | 0 | 0 | 0 |  | 0 | 0 |  | 0 | 0 |  |
| CD34 | Positive | 15 | 9 | 6 | 0.443 ^§^ | 0 | 15 | - | 1 | 14 | 0.553 ^§^ |
|  | Vessel positive | 13 | 5 | 8 |  | 0 | 13 |  | 1 | 12 |  |
|  | Negative | 34 | 15 | 19 |  | 0 | 34 |  | 1 | 33 |  |
|  | Missing | 24 | 9 | 15 |  | 1 | 23 |  | 0 | 24 |  |
| Ki67 | Positive rate ≥90% | 24 | 10 | 14 | 0.600 ^§^ | 1 | 23 | 0.279 ^§^ | 0 | 24 | 0.965 ^§^ |
|  | Positive rate 80%~90% | 25 | 9 | 16 |  | 0 | 25 |  | 2 | 23 |  |
|  | Positive rate 70%~80% | 19 | 12 | 7 |  | 0 | 19 |  | 1 | 18 |  |
|  | Positive rate 60%~70% | 14 | 5 | 9 |  | 0 | 14 |  | 0 | 14 |  |
|  | Positive rate 50%~60% | 1 | 1 | 0 |  | 0 | 1 |  | 0 | 1 |  |
|  | Positive rate <50% | 3 | 1 | 2 |  | 0 | 3 |  | 0 | 3 |  |
|  | Missing | 0 | 0 | 0 |  | 0 | 0 |  | 0 | 0 |  |
| P53 | Positive rate ≥90% | 27 | 13 | 14 | 0.215 ^§^ | 0 | 27 | 0.299 ^§^ | 0 | 27 | 0.327 ^§^ |
|  | Positive rate 80%~90% | 6 | 4 | 2 |  | 0 | 8 |  | 0 | 6 |  |
|  | Positive rate 70%~80% | 5 | 5 | 0 |  | 0 | 5 |  | 0 | 5 |  |
|  | Positive rate 60%~70% | 0 | 0 | 0 |  | 0 | 0 |  | 0 | 0 |  |
|  | Positive rate 50%~60% | 1 | 0 | 1 |  | 0 | 1 |  | 0 | 1 |  |
|  | Positive rate <50% | 20 | 5 | 15 |  | 0 | 20 |  | 2 | 18 |  |
|  | Negative | 26 | 10 | 16 |  | 1 | 25 |  | 1 | 25 |  |
|  | Missing | 1 | 1 | 0 |  | 0 | 1 |  | 0 | 1 |  |
| MLH1 | Positive | 73 | 31 | 42 | 0.538 ^§^ | 1 | 72 | 0.871 ^§^ | 1 | 72 | 0.112 ^§^ |
|  | Partially positive | 7 | 2 | 5 |  | 0 | 7 |  | 1 | 6 |  |
|  | Negative | 5 | 4 | 1 |  | 0 | 5 |  | 1 | 4 |  |
|  | Missing | 1 | 1 | 0 |  | 0 | 1 |  | 0 | 1 |  |
| MSH6 | Positive | 74 | 33 | 41 | 0.600 ^§^ | 1 | 73 | 0.871 ^§^ | 3 | 71 | 0.710 ^§^ |
|  | Partially positive | 5 | 2 | 3 |  | 0 | 5 |  | 0 | 5 |  |
|  | Negative | 6 | 2 | 4 |  | 0 | 6 |  | 0 | 6 |  |
|  | Missing | 1 | 1 | 0 |  | 0 | 1 |  | 0 | 1 |  |
| MSH2 | Positive | 76 | 35 | 41 | 0.161 ^§^ | 1 | 75 | 0.894 ^§^ | 2 | 74 | 0.473 ^§^ |
|  | Partially positive | 6 | 2 | 4 |  | 0 | 6 |  | 0 | 6 |  |
|  | Negative | 3 | 0 | 3 |  | 0 | 3 |  | 1 | 2 |  |
|  | Missing | 1 | 1 | 0 |  | 0 | 1 |  | 0 | 1 |  |

^†^ Chi-square test; ^‡^ Fisher’s exact test; ^§^ Mann-Whitney test.
