# Supplementary material for: Mutation Status and Immunohistochemical Correlation of KRAS, NRAS, and BRAF in 260 Chinese Colorectal and Gastric Cancers
Source: Front Oncol. 2018 Oct 26;8:487. doi: 10.3389/fonc.2018.00487 (PMC6212577; doi:10.3389/fonc.2018.00487)
Supplement: Table S2 — Immunohistochemistry characteristics according to KRAS/NRAS/BRAF gene mutation status in rectal cancer. [file Table_2.DOCX]

Supplementary Material

**Mutation status and immunohistochemical correlation of *KRAS*, *NRAS* and *BRAF* in 260 Chinese colorectal and gastric cancers**

Qiwei Yang^1^, Sibo Huo^2^, Yujie Sui^1^, Zhenwu Du^1,3^, Haiyue Zhao^4^, Yu Liu^2^, Wei Li^2^, Xin Wan^2^, Tongjun Liu^2*^, Guizhen Zhang^1,3*^

***Correspondence:** Professor. Guizhen Zhang: [zhangguizhenjlu@163.com](mailto:zhangguizhenjlu@163.com) & Professor. Tongjun Liu [tongjunliu@163.com](mailto:tongjunliu@163.com)

Table S2. Immunohistochemistry characteristics according to KRAS/NRAS/BRAF gene mutation status in rectal cancer.

|  |  | Total Case | KRAS (codon 12/13) | | | NRAS (codon 12/13/59/61/117/146) | | | BRAF (codon 600) | | |
| --- | --- | --- | --- | --- | --- | --- | --- | --- | --- | --- | --- |
|  |  | 140 | MT, n | WT, n | p vale | MT, n | WT, n | p vale | MT, n | WT, n | p vale |
| BRAF (V600E) | Positive | 4 | 1 | 3 | 0.626 ^†^ | 0 | 4 | 1.000 ^‡^ | 0 | 4 | - |
|  | Negative | 130 | 48 | 82 |  | 6 | 124 |  | 0 | 130 |  |
|  | Missing | 6 | 3 | 3 |  | 0 | 6 |  | 1 | 5 |  |
| PMS2 | Positive | 136 | 49 | 87 | 1.000 ^‡^ | 6 | 130 | 1.000 ^‡^ | 0 | 136 | - |
|  | Negative | 2 | 1 | 1 |  | 0 | 1 |  | 0 | 2 |  |
|  | Missing | 2 | 2 | 0 |  | 0 | 2 |  | 1 | 1 |  |
| EGFR | Positive | 62 | 20 | 42 | 0.198 ^§^ | 5 | 57 | **0.021** ^§^ | 0 | 62 | - |
|  | Weakly positive | 42 | 15 | 27 |  | 0 | 42 |  | 0 | 42 |  |
|  | Negative | 32 | 15 | 17 |  | 0 | 32 |  | 0 | 32 |  |
|  | Missing | 4 | 2 | 2 |  | 1 | 3 |  | 1 | 3 |  |
| CDX2 | Positive | 135 | 50 | 85 | 0.533 ^‡^ | 6 | 129 | 1.000 ^‡^ | 0 | 135 | - |
|  | Partially positive | 2 | 0 | 2 |  | 0 | 2 |  | 0 | 2 |  |
|  | Missing | 3 | 2 | 1 |  | 0 | 3 |  | 0 | 3 |  |
| CD34 | Positive | 14 | 7 | 7 | 0.908 ^§^ | 0 | 14 | 0.684 ^§^ | 0 | 14 | - |
|  | Vessel positive | 29 | 7 | 22 |  | 3 | 26 |  | 0 | 29 |  |
|  | Negative | 58 | 22 | 36 |  | 2 | 56 |  | 0 | 58 |  |
|  | Missing | 39 | 16 | 23 |  | 1 | 38 |  | 1 | 38 |  |
| Ki67 | Positive rate ≥90% | 51 | 14 | 37 | 0.269 ^§^ | 3 | 48 | 0.631 ^§^ | 0 | 51 | - |
|  | Positive rate 80%~90% | 44 | 20 | 24 |  | 1 | 43 |  | 0 | 44 |  |
|  | Positive rate 70%~80% | 28 | 11 | 17 |  | 2 | 26 |  | 0 | 28 |  |
|  | Positive rate 60%~70% | 9 | 4 | 5 |  | 0 | 9 |  | 0 | 9 |  |
|  | Positive rate 50%~60% | 5 | 1 | 4 |  | 0 | 5 |  | 0 | 5 |  |
|  | Positive rate <50% | 0 | 0 | 0 |  | 0 | 0 |  | 0 | 0 |  |
|  | Missing | 3 | 2 | 1 |  | 0 | 3 |  | 1 | 2 |  |
| P53 | Positive rate ≥90% | 63 | 19 | 44 | 0.504 ^§^ | 4 | 59 | 0.335 ^§^ | 0 | 63 | - |
|  | Positive rate 80%~90% | 11 | 6 | 5 |  | 0 | 11 |  | 0 | 11 |  |
|  | Positive rate 70%~80% | 2 | 0 | 2 |  | 0 | 2 |  | 0 | 2 |  |
|  | Positive rate 60%~70% | 3 | 1 | 2 |  | 0 | 3 |  | 0 | 3 |  |
|  | Positive rate 50%~60% | 4 | 3 | 1 |  | 1 | 3 |  | 0 | 4 |  |
|  | Positive rate <50% | 16 | 9 | 7 |  | 0 | 16 |  | 0 | 16 |  |
|  | Negative | 38 | 12 | 26 |  | 1 | 37 |  | 0 | 38 |  |
|  | Missing | 3 | 2 | 1 |  | 0 | 3 |  | 1 | 2 |  |
| MLH1 | Positive | 120 | 43 | 77 | 0.641 ^§^ | 5 | 115 | 0.754 ^§^ | 0 | 120 | - |
|  | Partially positive | 16 | 6 | 10 |  | 1 | 15 |  | 0 | 16 |  |
|  | Negative | 1 | 1 | 0 |  | 0 | 1 |  | 0 | 1 |  |
|  | Missing | 3 | 2 | 1 |  | 0 | 3 |  | 1 | 2 |  |
| MSH6 | Positive | 119 | 41 | 78 | 0.192 ^§^ | 5 | 114 | 0.801 ^§^ | 0 | 119 | - |
|  | Partially positive | 17 | 8 | 9 |  | 1 | 16 |  | 0 | 17 |  |
|  | Negative | 1 | 1 | 0 |  | 0 | 1 |  | 0 | 1 |  |
|  | Missing | 3 | 2 | 1 |  | 0 | 3 |  | 1 | 2 |  |
| MSH2 | Positive | 126 | 45 | 81 | 0.521 ^§^ | 5 | 121 | 0.428 ^§^ | 0 | 126 | - |
|  | Partially positive | 11 | 5 | 6 |  | 1 | 10 |  | 0 | 11 |  |
|  | Negative | 0 | 0 | 0 |  | 0 | 0 |  | 0 | 0 |  |
|  | Missing | 3 | 2 | 1 |  | 0 | 3 |  | 1 | 2 |  |

^†^ Chi-square test; ^‡^ Fisher’s exact test; ^§^ Mann-Whitney test.
